# Clinical Diagnosis of Temporal Arteritis With Seronegative and Negative Biopsy Studies

**DOI:** 10.7759/cureus.31011

**Published:** 2022-11-02

**Authors:** Angel Bayas, Octavio Carranza, Marc A Swerdloff

**Affiliations:** 1 Neurology, Florida Atlantic University Charles E. Schmidt College of Medicine, Boca Raton, USA; 2 Marcus Neuroscience Institute, Boca Raton Regional Hospital, Boca Raton, USA

**Keywords:** giant cell arteritis, temporal arteritis, erythrocyte sedimentation rate, c-reactive protein, c –reactive protein (crp), erythrocyte sedimentation rate (esr), temporal artery biopsy, temporal artertitis, giant cell arteritis (gca)

## Abstract

Giant cell arteritis (GCA), also known as temporal arteritis is the most common of the systemic vasculitides. It occurs in individuals older than 50 years of age and peaks in incidence in the seventh decade. The gold standard for diagnosis of GCA is temporal artery biopsy (TABx) which will show transmural inflammation, but a negative biopsy does not rule out the disease. We present a case of a 66-year-old male with a classic clinical presentation of temporal arteritis with a normal erythrocyte sedimentation rate (ESR), C-reactive protein (CRP), and negative bilateral temporal artery biopsies. He was discharged on prednisone. Ten days later, he unilaterally stopped his corticosteroid treatment leading to a recurrence of symptoms and conversion to seropositivity (ESR negative and CRP positive). The objective of this article is to point out that the diagnosis of temporal arteritis is clinical and is not discarded by a negative TABx. Patients with classic clinical manifestations of temporal arteritis but with a negative TABx should be treated aggressively.

## Introduction

Giant cell arteritis (GCA), also known as temporal arteritis is the most common of the systemic vasculitides. Temporal artery biopsy (TABx) is the gold standard for diagnosis of GCA with a sensitivity of 15%-40% and a specificity of 100%. Unfortunately, TABx has a false negative rate as high as 7% [1]. Elevation in the erythrocyte sedimentation rate (ESR) and C-reactive protein (CRP) are variables for diagnosis criteria of GCA but may be normal in 7%-20% of patients with GCA [2]. 

The American College of Rheumatology (ACR), in 1990 created a criterion for the diagnosis of GCA in the "Traditional Format." Later the ACR presented the Tree format that used the same traditional format, except that ESR was excluded, and two other variables were included [3]. The last revision from the ACR for diagnosis criteria of GCA, (rACR) was published in 2016. This revision contains 11 points (p). It proposed that in the existence of three points or more, with at least one point included in Domain 1, the diagnosis of GCA can be accepted, however, this is yet to be evaluated [4].

The objective of this article is to point out that the diagnosis of temporal arteritis is clinical and is not discarded by a negative TABx. Patients with classic clinical manifestations of temporal arteritis but with a negative TABx should be treated aggressively. 

## Case presentation

A 66-year-old male presented to the emergency room complaining of a right temporal headache, difficulty with balance, and blurry vision. The headache woke him from sleep two days in a row. It was intermittent throughout the day. He never experienced headaches prior to this. He did not take medication to alleviate this headache. The day before his presentation, he experienced transient vertigo with diaphoresis, nausea, and heaviness in his lower extremities. He felt off balance while walking. He had jaw claudication. He denied falls, loss of consciousness, fevers, or chills. He was initially treated as a stroke alert; however, the duration of symptoms and an NIHSS score of 0 made acute stroke intervention unlikely. On physical examination, his visual fields were intact. Pupils were equally round and reactive to light to direct and indirect light bilaterally. Extraocular movements were intact. No nystagmus was noted. The facial sensation was intact. The face was symmetric. No dysarthria. MRI and CT scan of the head was negative. Laboratory values are listed in Table 1.

**Table 1:** Laboratory values. BUN, blood urea nitrogen; GFR, glomerular filtration rate; ALT, alanine transaminase; AST, aspartate transaminase; WBC, white blood cell; RBC, red blood cell; HGB, hemoglobin; HCT, hematocrit; PLT, platelet count; INR, international normalized ratio; ESR, erythrocyte sedimentation rate; CRP, C-reactive protein; PTT, partial thromboplastin time; PT, prothrombin time

Test	Result	Normal range	Test	Result	Normal range
WBC	7.900 K/mcL	4.1–10.9 K/mcL	Na	139 mmol/L	138–148 mmol/L
RBC	4.71 mcL	4.20–6.30 mcL	K	3.7 mmol/L	3.6–5.2 mmol/L
HGB	15.0 g/dL	12.0–18.0 g/dL	Glucose	106 mg/dL	70–99 mg/dL
HCT	44.5%	37.0%–51.0%	BUN	21 mg/dL	7–18 mg/dL
PLT	211 K/mcL	140–440 K/mcL	Creatinine	1.3 mg/dL	0.6–1.3 mg/dL
ESR	(12) mm/h (N)	0–20 mm/h	GFR	59 mL/min/1,73 m2	>59 mL/min/1.73
CRP	0,39 mg/dL	<0.80 mg/dL	AST	24 Int/Unit/L	10–31 Int/Unit/L
INR: PT	1.0/136 s	0.9–1.2/11.5–14.4 s	ALT	37 Int/Unit/L	12–78 Int/Unit/L
PTT	30.9 s	22–34.8 s	Alkaline phosphatase	41 Int/Unit/L	45–117 Int/Unit/L

A vascular surgery consultation refused to perform a biopsy citing the low yield of a positive biopsy in a seronegative setting. A rheumatology consultation concluded the case was compelling for GCA and recommended long-term steroids even if a TABx was negative. The vascular neurosurgical team performed a bilateral TABx. The pathology report was negative for bilateral temporal arteritis.

After one dose of prednisone, he was able to sleep uninterrupted by headache. His other symptoms also ceased. He was sent home on 60 mg prednisone daily. Ten days later, he presented to the hospital after abruptly stopping his medication after a week of treatment. He developed chills, dizziness, numbness in his hands, and slight blurring in his distance vision. He returned to the hospital where a CRP was elevated to 4 mg/dL.

Ophthalmology evaluation described floaters in both eyes. Visual acuity with correction was oculus sinister (OS): 20/40 and oculus dextrus (OD): 20/40. Visual fields were intact to finger confrontation. No dyschromatopsia on Ishihara testing oculus uterque (OU). Pupils were equally round and reactive to light bilaterally. There was no afferent pupillary defect (APD). Extraocular movements were intact without nystagmus. Fundoscopic examination was normal. He was discharged home on 60 mg prednisone daily.

## Discussion

The American College of Rheumatology (ACR), in 1990 created a criterion for the diagnosis of GCA the "Traditional Format." In this format, the presence of three or more of these five variables can suggest the diagnosis of GCA and was associated with a sensitivity of 93.5% and a specificity of 91.2%. See Table 2, for the traditional format for the diagnosis of GCA [3]. 

**Table 2:** 1990 Criteria for the classification of GCA (Traditional format). ESR, erythrocyte sedimentation rate; GCA, giant cell arteritis Reference: The American College of Rheumatology

Age at disease onset >= 50 years. New onset of or new type of localized headache. Temporal artery abnormality (tenderness to palpation or decreased temporal artery pulse). Elevated ESR greater than or equal to 50 mm/h abnormal biopsy of an artery.

Later the ACR presented the Tree format that used the same traditional format, except that ESR was excluded, and two other variables were included: scalp tenderness and claudication of the jaw or tongue or on deglutition. The Tree format was associated with a sensitivity of 95.3% and a specificity of 90.7%, as listed in Table 3 [3].

**Table 3:** Criteria and definition used for the classification of GCA (Tree format). GCA, giant cell arteritis Reference: The American College of Rheumatology

Criterion	Definition
Age at disease onset >= 50 years	Development of symptoms or findings beginning at age 50 or older
New headache	New onset of or a new type of localized headache
Claudication of jaw, tongue, or on deglutition	Temporal artery abnormality (tenderness to palpation or decreased temporal artery pulse, unrelated to arteriosclerosis of cervical arteries
Scalp tenderness or nodules	Development of tenderness areas or nodules over the scalp, away from the temporal artery or other cranial arteries
Abnormal artery biopsy	Biopsy specimen with artery showing vasculitis characterized by a predominance of mononuclear cell infiltration or granulomatous inflammation, usually with multinucleated giant cells

The last revision from the ACR for diagnosis criteria of GCA, (rACR) was published in 2016. This revision contains 11 points (p) as listed in Table 4. It proposed that in the existence of three points or more, with at least one point included in Domain 1, the diagnosis of GCA can be accepted, however, this is yet to be evaluated [4].

**Table 4:** Revised ACR criteria (rACR) for diagnosis of GCA. rACR, revision for American College of Rheumatology; ESR, erythrocyte sedimentation rate; PMR, polymyalgia rheumatica; GCA, giant cell arteritis Reference: International Journal of Surgery Open, Vol 9., 2017, pp. 19-23

Score	Entry
N/A	Age at onset >50 years old absence of exclusion criteria
	Domain I
1	New onset localized headache
1	Sudden onset of visual disturbances
2	PMR
1	Jaw claudication
2	Abnormal temporal artery
	Domain II
1	Unexplained fever and/or anemia
1	ESR > 50 mm/h
2	Compatible pathology

Our case meets the age criteria, absence of exclusion criteria, and in Domain I, new onset of localized headache, sudden onset of visual disturbance, and jaw claudication.

Table 5 listed the comparison of the sensitivity and specificity of potential criteria variables for GCA [3].

**Table 5:** Comparison of the sensitivity and specificity of potential criteria variables for GCA. GCA, giant cell arteritis; PMR, polymyalgia rheumatica; TA, temporal artery Reference: The American Academy of Rheumatology

Criterion	#. Of patients (n=214)	#. Of controls (n=593)	Sensitivity	Specificity
History				
Age at disease onset >=50 years	213	588	98.6	63.8
Headache, new, localized	214	590	64.5	81.9
Jaw claudication	213	579	38.5	97.9
Tongue claudication	213	578	2.8	99.8
Claudication on deglutition	212	577	4.2	99.0
Claudication, variables 3-5	212	576	40.6	97.6
AM stiffness neck/torso	211	586	50.2	86.5
AM stiffness shoulders/arms	210	586	52.9	77.5
AM stiffness hips/thighs	211	586	46.9	79.7
PMR	210	585	52.9	79.3
Diplopia	213	587	11.3	93.9
Physical				
Ischemic optic neuritis	212	580	7.5	98.4
Amaurosis fugax	214	588	11.2	95.7
Partial unilateral loss of vision	213	587	4.2	99.0
Complete unilateral loss of vision	212	585	3.3	99.7
Partial bilateral loss of vision	214	584	2.3	99.1
Optic atrophy	212	586	4.7	99.0
Visual abnormality	210	573	27.6	88.8
Right TA tenderness	212	495	23.1	99.6
Decrease right TA pulse	210	477	35.2	97.9
Left TA tenderness	211	494	21.3	99.2
Decrease left TA pulse	209	476	28.7	97.9
TA abnormality	211	473	57.3	96.8
Scalp tenderness	212	584	40.6	97.9
Scalp nodules	212	585	13.7	99.5
Scalp tenderness or nodules	212	581	43.9	97.4

Studies showed that ESR, CRP, and platelets are moderate, equivalent diagnostic tests for GCA [5]. These studies can help to guide the GCA diagnosis, but they are not specific to the diagnosis. Approximately four percent of patients have normal ESR and CRP values [6].

The gold standard for diagnosis of GCA is TABx with or without temporal artery color Doppler ultrasound (CDUS). TABx should be performed in any patient with suspected temporal arteritis. The treatment with systemic corticosteroids should not be delayed until after TAB is done [7]. TABx has a specificity of 100%, but poor sensitivity when compared with clinical diagnosis with a false-negative rate reported as high as 44% [8]. The characteristic histologic finding of GCA is transmural inflammatory infiltrate of lymphocytes, macrophages, and, in 75% of cases, giant cells [1].

The following are reasons for a high false negativity rate in TABx:

1.. Skip lesions: The pathology is segmental, and a biopsy may miss the area of involvement.

2. Length of specimen: With non-continuous pathology, the lesion cannot be localized a longer biopsy specimens and bilateral sites are recommended [9].

3. Number of sections evaluated: In order not to not miss inflammatory changes, at least three additional slices should be evaluated in all negative TABx specimens [9].

4. Effect of therapy: Glucocorticoid treatment may reduce the sensitivity of TABx. Resolution of the inflammatory infiltrate of GCA occurs slowly, and histopathologic evidence will be evident for at least a month after glucocorticoid therapy has.

The most common complications of TABx are bleeding, surgical site infection, nerve injury involving the auriculotemporal nerve or branches of the facial nerve. In a series of 75 biopsies performed on 68 patients, 16% of patients developed new frontalis muscle weakness with complete recovery after one year (two cases) [10]. A rare complication of TABx seen by one of us (M Swerdloff), was an operating room fire triggered by electrocautery applied to a flammable local disinfectant. The patient was transferred to a regional burn unit with burns to her scalp. She was lost to follow up. 

Other noninvasive vessel wall imaging to complement the diagnosis of GCA such as high-resolution contrast enhanced magnetic resonance angiography (MRA) may identify mural edema in the temporal artery [11]. There is evidence supporting the use of Doppler ultrasound, dedicated post-contrast T1-weighted spin echo MRI of the scalp arteries, and positron emission tomography (PET) scan. These studies can improve diagnostic accuracy of GCA in cases in which TABx is inconclusive or negative [12]. In our case we did not use these studies due to the strong clinical findings that our patient presented. 

Some of the differential diagnoses to consider are Takayasu arteritis, small- and medium-sized vessel vasculitides, primary angiitis of the central nervous system, idiopathic aortitis, non-arteritic anterior ischemic optic neuropathy, and infection [13].

Steroid treatment should not be delayed awaiting TABx results. The 2016 revised ACR criteria (rACR) help us to have strong guidance for the GCA diagnosis in patients who meet the criteria. Clinical improvement after high-dose glucocorticoids is nonspecific and should not be the sole reason for establishing the diagnosis of GCA but it is supportive. Close follow-up to monitor the clinical course of the disease and to mitigate long-term corticosteroid treatment side effects is necessary.

## Conclusions

The 2016 rACR criteria for the diagnosis of GCA are still in review. New imaging strategies such as temporal ultrasound, MRI, MRA, and PET scans may reinforce the diagnosis. There remain economic barriers to instituting all these modalities. The objective of this article is to point out that the diagnosis of temporal arteritis is clinical and is not discarded by a negative TABx. Patients with classic clinical manifestations of temporal arteritis but with a negative TABx should be treated aggressively. The treating physicians should follow clinical symptoms and signs during the tapering process when serological guidance is not present. We recommend a rheumatology consultation to support a full workup and treatment when disagreements arise among the treating specialties.
